# Comparative and phylogenetic analysis of chloroplast genomes from four species in *Quercus* section *Cyclobalanopsis*

**DOI:** 10.1186/s12863-024-01232-y

**Published:** 2024-06-10

**Authors:** Buyu Li, Ke Huang, Xiaoli Chen, Chun Qin, Xuemei Zhang

**Affiliations:** College of Life Science, China West Normal University, Nanchong, 637000 China

**Keywords:** Chloroplast (cp) genome, Phylogenetic analysis, *Quercus blakei*, *Quercus dinghuensis*, *Quercus disciformis*, *Quercus hui*

## Abstract

The *Quercus* L. species is widely recognized as a significant group in the broad-leaved evergreen forests of tropical and subtropical East Asia. These plants hold immense economic value for their use as firewood, furniture, and street trees. However, the identification of *Quercus* species is considered challenging, and the relationships between these species remain unclear. In this study, we sequenced and assembled the chloroplast (cp.) genomes of four *Quercus* section *Cyclobalanopsis* species (*Quercus disciformis*, *Quercus dinghuensis*, *Quercus blackei*, and *Quercus hui*). Additionally, we retrieved six published cp. genome sequences of *Cyclobalanopsis* species (*Quercus fleuryi*, *Quercus pachyloma*, *Quercus ningangensis*, *Quercus litseoides*, *Quercus gilva*, and *Quercus myrsinifolia*). Our aim was to perform comparative genomics and phylogenetic analyses of the cp. whole genome sequences of ten *Quercus* section *Cyclobalanopsis* species. The results revealed that: (1) *Quercus* species exhibit a typical tetrad structure, with the cp. genome lengths of the newly sequenced species (*Q. disciformis*, *Q. dinghuensis*, *Q. blakei*, and *Q. hui*) being 160,805 bp, 160,801 bp, 160,787 bp, and 160,806 bp, respectively; (2) 469 SSRs were detected, among which A/T base repeats were the most common; (3) no rearrangements or inversions were detected within the chloroplast genomes. Genes with high nucleotide polymorphism, such as *rps14-psaB*, *ndhJ-ndhK*, *rbcL-accD*, and *rps19-rpl2_2*, provided potential reference loci for molecular identification within the *Cyclobalanopsis* section; (4) phylogenetic analysis showed that the four sections of *Cyclobalanopsis* were grouped into sister taxa, with *Q. hui* being the first to diverge from the evolutionary branch and *Q. disciformis* being the most closely related to *Q. blackei*. The results of this study form the basis for future studies on taxonomy and phylogenetics.

## Introduction

The genus *Quercus*, also known as oaks, is the largest genus within the *Fagaceae* family, comprising more than 500 species [38]. It has long been closely associated with human ecology [17, 56]. With a wide distribution in tropical, temperate, and semi-arid regions, this genus is considered a key species in the Northern Hemisphere and a dominant component of subtropical broad-leaved forests. Additionally, its wood is highly valued for its hardness and beautiful grain, making it a high-quality material for construction, furniture, and even mushroom cultivation [7, 27]. The classification of the genus *Quercus* is challenging due to its complex evolutionary history, and it remains an ongoing endeavor [12, 28, 37]. To address this, a novel global phylogenetic framework has been developed for the genus *Quercus*, using nuclear molecular markers and pollen characteristics. This framework divides the genus into two subgenera, *Quercus* and *Cerris*, each further divided into eight Sect [14]. China includes all four previously recognized sections: *Quercus*, *Cerris*, *Cyclobalanopsis*, and *Ilex* [19, 53]. However, the representation of Chinese species within this framework is limited and much smaller compared to that of North American and European species.

The traditional classification of the genus *Quercus* has traditionally relied on morphological features, epidermal characteristics of leaves, pollen studies, and the evolutionary processes of a limited number of species [11, 13, 42, 44]. However, molecular sequence data have shown discrepancies with the groups identified through traditional morphological classifications within oaks [14]. For example, research based on ITS sequences suggests that the species of the compound trichome base (CTB) group in the *Quercus* section *Cyclobalanopsis* cluster together with the *Quercus* section *Cerris*, which significantly deviates from the traditional morphological classification. This lack of consensus on the classification of the genus *Quercus* indicates the need for further molecular evidence to understand the interspecific relationships and infrageneric phylogenetic status within the *Quercus* genus, particularly considering the similarities in leaf characteristics and gene introgression among different groups.

Chloroplasts are semi-autonomous organelles responsible for photosynthesis and the synthesis of various compounds such as amino acids, pigments, starch, and fatty acids. Previous research has shown that these organelles have their own genetic system [5, 43]. In angiosperms, the composition, arrangement, and configuration of chloroplast chromosomes are often conserved across different plant taxa. However, intraspecific variation can occur, leading to gene and intron gains and losses, as well as expansions, contractions, and inversions at the inverted repeat boundaries. Comparative analysis of chloroplast chromosomes provides valuable material for studying plant lineages and evolution. Moreover, the chloroplast genome has emerged as a more reliable source of genomic material for studying evolutionary relationships and intraspecific diversity compared to traditional taxonomic techniques [35].

Following the initial release of the *Quercus rubra* cp. genome in 2014, there has been a notable surge in the sequencing of cp. genomes across various oak species. This surge exemplifies the rapid advancements in second-generation sequencing technology and the growing utilization of cp. genomes for reconstructing phylogenetic relationships [1, 23, 55, 57]. To date, only 16 cp. genomes from the section *Cyclobalanopsis*, a subgenus of *Quercus*, have been characterized. This limitation hampers the application of phylogenetic analysis and molecular identification within the *Quercus* genus. To resolve taxonomic uncertainties in *Quercus*, further investigation of cp. genome data is necessary.

In this study, we assembled, spliced, and annotated the complete cp. genomes of four *Quercus* section *Cyclobalanopsis* species. We then compared them with the previously published cp. genomes of six other subgenera from GenBank. Our specific objectives were to: (a) fully assemble and annotate the structure and functional genes of the cp. genomes in section *Cyclobalanopsis*; (b) analyze codon usage; (c) identify tandem repeats, random repeats, and mutated regions in section *Cyclobalanopsis* that contribute to species identity and evolution; (d) infer phylogenetic relationships among species in section *Cyclobalanopsis* using the entire cp. genome.

## Results

### Basic properties of the cp. genome

**Fig. 1:**
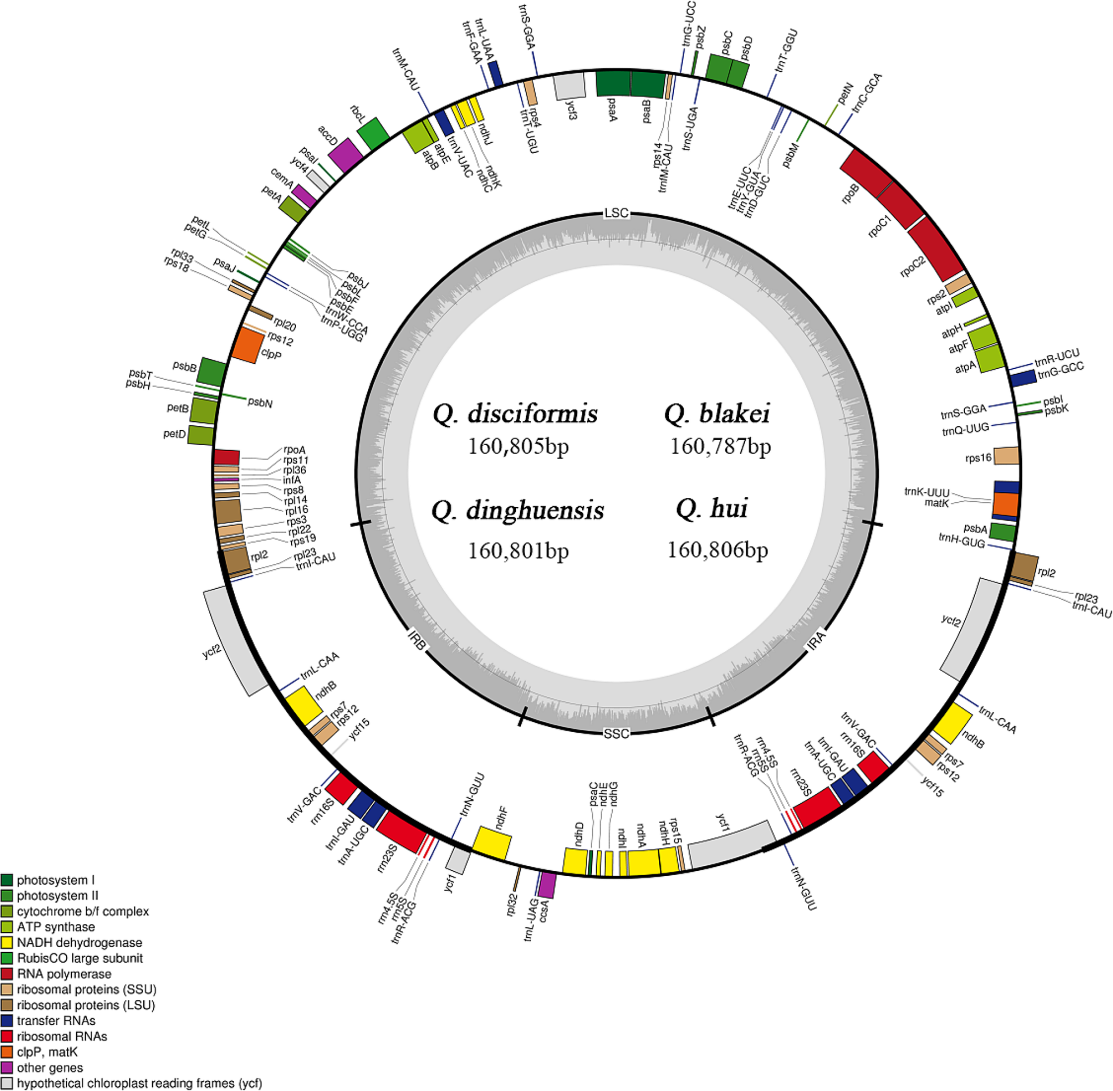
Gene map of four *Quercus* section *Cyclobalanopsis* species. Genes inside and outside the circle are transcribed clockwise and counterclockwise separately. Darker and lighter grey in the inner circle each represent GC and AT content

We successfully sequenced the complete cp. genomes of four *Cyclobalanopsis* species, with lengths ranging from 160,787 to 160,806 base pairs (Fig. 1). The gene composition, sequence, and arrangement were remarkably conserved among these cp. genomes. These genomes exhibit the typical quadripartite structure found in most angiosperms, with a LSC region (90,201 bp to 90,276 bp) flanked by two IR regions (25,811 bp to 25,842 bp), separating it from the SSC region (18,877 bp to 18,908 bp). Notably, there is a 19 bp difference in length between *Q. hui* and *Q. blakei*, with *Q. hui* being the longer sequence (Tables 1 and 2). In total, 132 genes were pinpointed, encompassing 8 rRNA genes, 37 tRNA genes, and 87 protein-coding genes (Table 3). The coding genes were primarily composed of self-replication genes, photosynthesis genes, ycf genes, and “other” genes. Fourteen genes contain a single intron (*trnA-UGC*, *trnG-GCC*, *trnI-GAU*, *trnK-UUU*, *trnL-UAA*, *trnV-UAC*, *rps16*, *rpl2*, *rpoC1*, *ndhA*, *ndhB*, *petB*, *petD*, *and atpF*), while four genes have two introns (*ycf3*, *clpP*, *ycf15*, and *rps12*).

**Table 1:** Comparison of cp. genomes among four species of section *Cyclobalanopsis*

Plant name	Collection place	Latitude/Longitude	Time of collection	Voucher specimen
*Quercus disciformis*	South China Botanical Garden, Chinese Academy of Sciences	E113.3672 N23.1818	2022.10.18	LY2210182
*Quercus dinghuensis*	South China Botanical Garden, Chinese Academy of Sciences	E109.0910 N19.1211	2022.10.18	LY2210181
*Quercus blakei*	Nanning Arboretum, Guangxi Province, China	E108.3135 N22.7094	2022.9.27	LY2209273
*Quercus hui*	Nanning Arboretum, Guangxi Province, China	E108.3135 N22.7094	2022.9.27	LY2209278

**Table 2:** A summary of the statistics for the cp. genomes of 4 *Quercus* sect. *Cyclobalanopsis* species

Species	Q. disciformis	Q. dinghuensis	Q. blakei	Q. hui
Genome size (bp)	160,805	160,801	160,787	160,806
Length of LSC (bp)	90,244	90,236	90,201	90,276
Lengh of IRB (bp)	25,842	25,842	25,842	25,811
Length of SSC (bp)	18,877	18,881	18,902	18,908
Lengh of IRA (bp)	25,842	25,842	25,842	25,811
Number of genes	132	132	132	132
protein-coding genes	87	87	87	87
tRNA genes	37	37	37	37
rRNA genes	8	8	8	8
GC content (%)	36.90	36.90	36.90	36.88
GC content of LSC (%)	34.74	34.74	34.75	34.73
GC content of IRB (%)	42.77	42.77	42.77	42.79
GC content of SSC (%)	31.12	31.12	31.10	31.06
GC content of IRA (%)	42.77	42.77	42.77	42.79

**Table 3:** Complete chloroplast genome map of section *Cyclobalanopsis*

	Gene group	Gene name
Self replication	Ribosomal RNA genes	*rrn16S*^*c*^,*rrn23S*^*c*^,*rrn4.5S*^*c*^,*rrn5S*^*c*^
	Transfer RNA genes	*trnA-UGC*^*bc*^,*trnC-GCA, trnD-GUC, trnE-UUC, trnF-GAA, trnG-GCC*^*b*^,*trnG-UCC*
		*trnH-GUG, trnI-CAU*^*c*^,*trnI-GAU*^*bc*^,*trnK-UUU*^*b*^,*trnL-CAA*^*c*^,*trnL-UAA*^*b*^,*trnL-UAG*
		*trnM-CAU*^*c*^,*trnN-GUU*^*c*^,*trnP-UGG, trnQ-UUG, trnR-ACG*^*c*^,*trnR-UCU, trnS-GGA*^*c*^
		*trnS-UGA, trnT-GGU, trnT-UGU, trnV-GAC*^*c*^,*trnV-UAC*^*b*^,*trnW-CCA, trnY-GUA*
		*trnfM-CAU*
	Small subunit of ribosome	*rps11,rps12*^*ac*^,*rps14,rps15,rps16*^*b*^,*rps18,rps19,rps2,rps3,rps4,rps7*^*c*^,*rps8*
	Large subunit of ribosome	*rpl14,rpl16,rpl2*^*bc*^,*rpl20,rpl22,rpl23*^*c*^,*rpl32,rpl33,rpl36*
	RNA polymerase subunits	*rpoA, rpoB, rpoC1*^*b*^,*rpoC2*
Photosynthesis	NADH dehydrogenase	*ndhA*^*b*^,*ndhB*^*bc*^,*ndhC, ndhD, ndhE, ndhF, ndhG, ndhH, ndhI, ndhJ, ndhK*
	Photosystem I	*psaA, psaB, psaC, psaI, psaJ*
	Photosystem II	*psbA, psbB, psbC, psbD, psbE, psbF, psbH, psbI, psbJ, psbK, psbL, psbM, psbN, psbT, psbZ*
	Cytochrome b/f complex	*petA, petB*^*b*^,*petD*^*b*^,*petG, petL, petN*
	ATP synthase	*atpA, atpB, atpE, atpF*^*b*^,*atpH, atpI*
	Rubisco	*rbcL*
Other genes	Maturase	*matK*
	Envelope membrane protein	*cemA*
	Subunit of acetyl-CoA-carboxylase	*accD*
	c-type cytochrome synthesis gene	*ccsA*
	Protease	*clpP* ^*a*^
	Tanslational initiation factor	*infA*
Genes of unknown function	Conserved open reading frames	*ycf1*^*c*^,*ycf2*^*c*^,*ycf3*^*a*^,*ycf4,ycf15*^*ac*^

^a^Gene with two introns. ^b^Gene with one intron. ^c^Genes located in the inverted repeats

### Codon usage

**Fig. 2:**
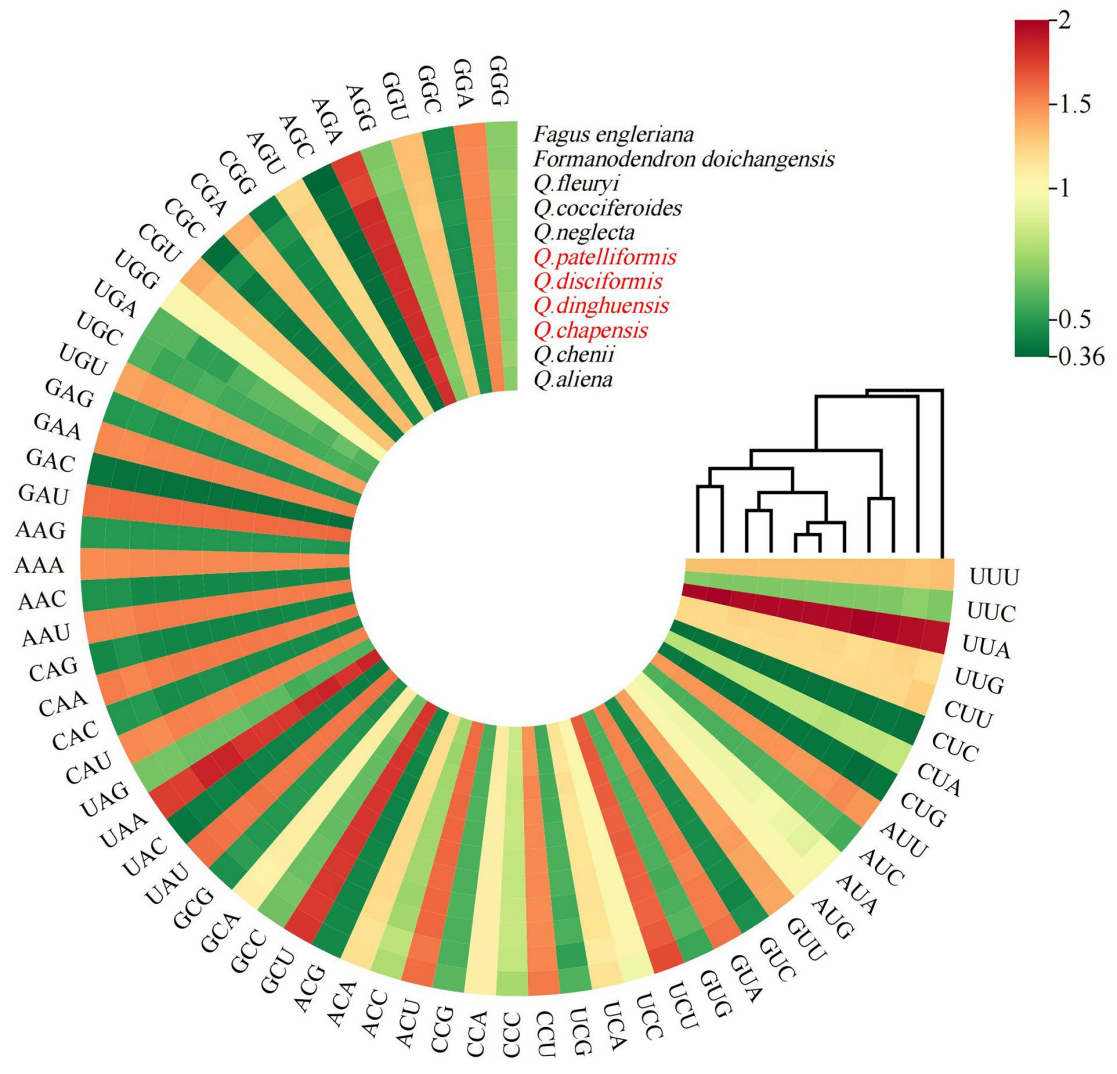
The RSCU of amino acids in 10 section *Cyclobalanopsis* cp. genome. Boxes below the graphs represent all codons encoding each amino acid. The colors of the histograms correspond to the colors of the codons

The codon usage patterns are summarized in Table 4. Protein-coding genes, ranging from 62,829 to 63,036 bp, were extracted from four species within the section. The counts of synonymous codons varied from 20,051 to 20,117, with an effective number of codons (ENC) close to 49.94 and a codon adaptation index (CAI) of 0.167. The CBI for *Q. disciformis*, *Q. dinghuensis*, *Q. hui*, and *Q. blakei* showed slight variations, ranging between − 0.099 and − 0.100. The frequency of optimal codons (FOP) for the four species ranged from 0.334 to 0.355, with GC content varying between 37.94% and 37.98%. Analysis of codon usage preference in the *Cyclobalanopsis* cp. genomes revealed 30 high-frequency codons with Relative Synonymous Codon Usage (RSCU) values greater than 1 (e.g., UAA, CCU, GCU), of which 28 ended with A or U bases and only two with G or C bases, indicating a bias towards A/U endings in the *Cyclobalanopsis* cp. genomes. However, the tryptophan codon (UGG) and the methionine codon (AUG) did not show a clear preference based on their RSCU values. Figure 2 demonstrates significant conservation of codon usage within the section *Cyclobalanopsis*, despite the presence of species-specific variations.

**Table 4:** Codon preference indices of 4 species of section *Cyclobalanopsis*

Index	Q. disciformis	Q. dinghuensis	Q. blakei	Q. hui
Length (bp)	63,036	63,036	62,853	62,829
Number of synonymous codons	20,117	20,117	20,060	20,051
Effective number of codons	49.94	49.94	49.95	49.94
Codon adaptation index	0.167	0.167	0.167	0.167
Codon bias index	-0.099	-0.099	-0.100	-0.099
Frequency of optimal codons	0.355	0.355	0.354	0.354
GC content (%)	37.94	37.95	37.98	37.97
GC1 content (%)	46.11	46.12	46.18	46.16
GC2 content (%)	37.83	37.83	37.83	37.83
GC3 content (%)	29.89	29.89	29.92	29.92

### Comparative analysis of cp. genes

#### Repeat sequences and SSR

**Fig. 3:**
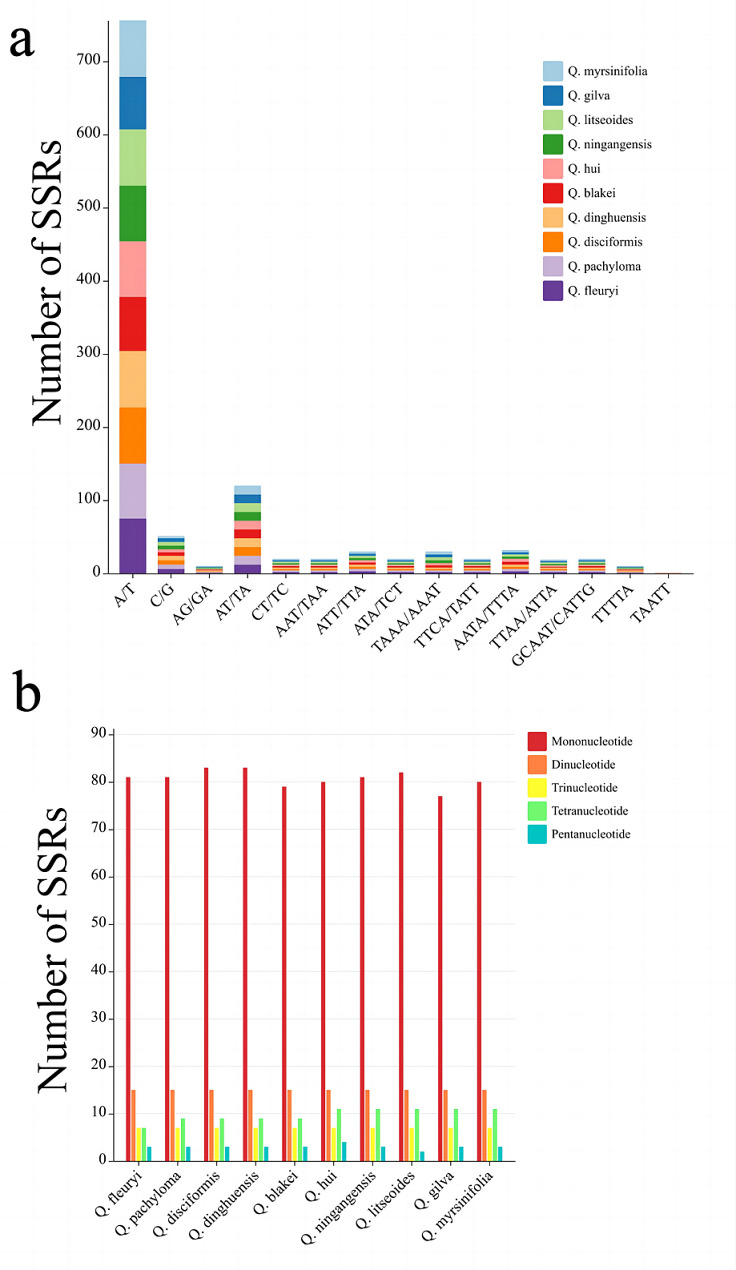
SSR type and number of cp. genome in 10 section *Cyclobalanopsis* cp. genome

**Fig. 4:**
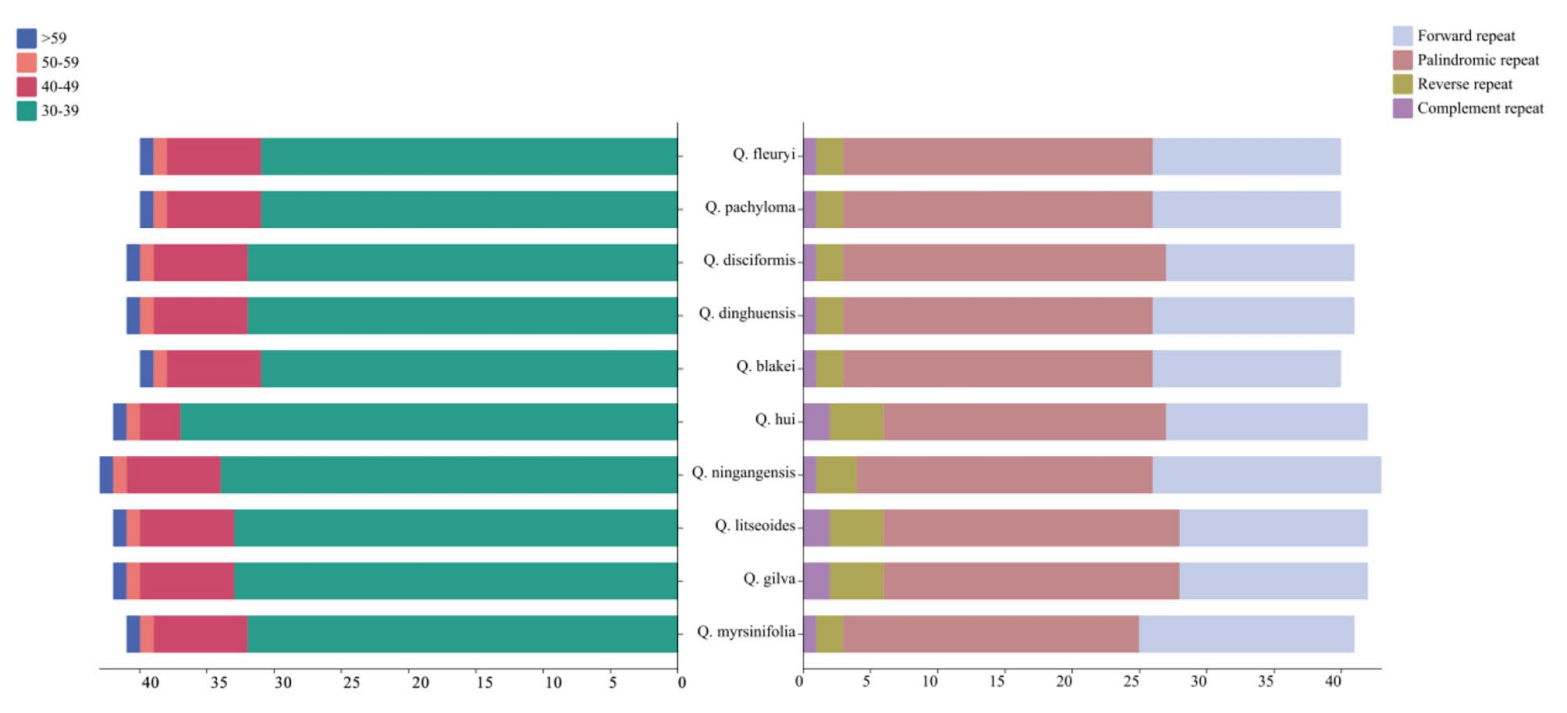
Scattered repeats type and number of cp. genome in 10 section *Cyclobalanopsis* cp. genome

The SSR analysis identified five categories of SSRs: single, dinucleotide, trinucleotide, tetranucleotide, and pentanucleotide repeats (Fig. 3b). Notably, hexanucleotide SSRs were absent. The chloroplast genomes of ten plant species showed a predominance of mononucleotide SSRs, with pentanucleotide SSRs being the least frequent. The *Q. litseoides* region had the highest number of SSRs (118), while *Q. blakei* had the lowest (113). The variation in SSR counts among the species was not statistically significant. In the ten *Cyclobalanopsis* species, A/T simple repeats were the most common, with AG/GA and TTTTA repeats occurring only once each. Uniquely, the SSR TAATT was identified solely in *Q. hui* (Fig. 3a). The search for dispersed repetitive sequences revealed four types: palindromic (P), complement (C), reverse (R), and forward (F). Although there were slight variations in their numbers (ranging from 40 to 43), palindromic repeats (P) were the most prevalent, while complementary repeats (C) were the least common (Fig. 4).

#### Expansion and contraction of the border regions

**Fig. 5:**
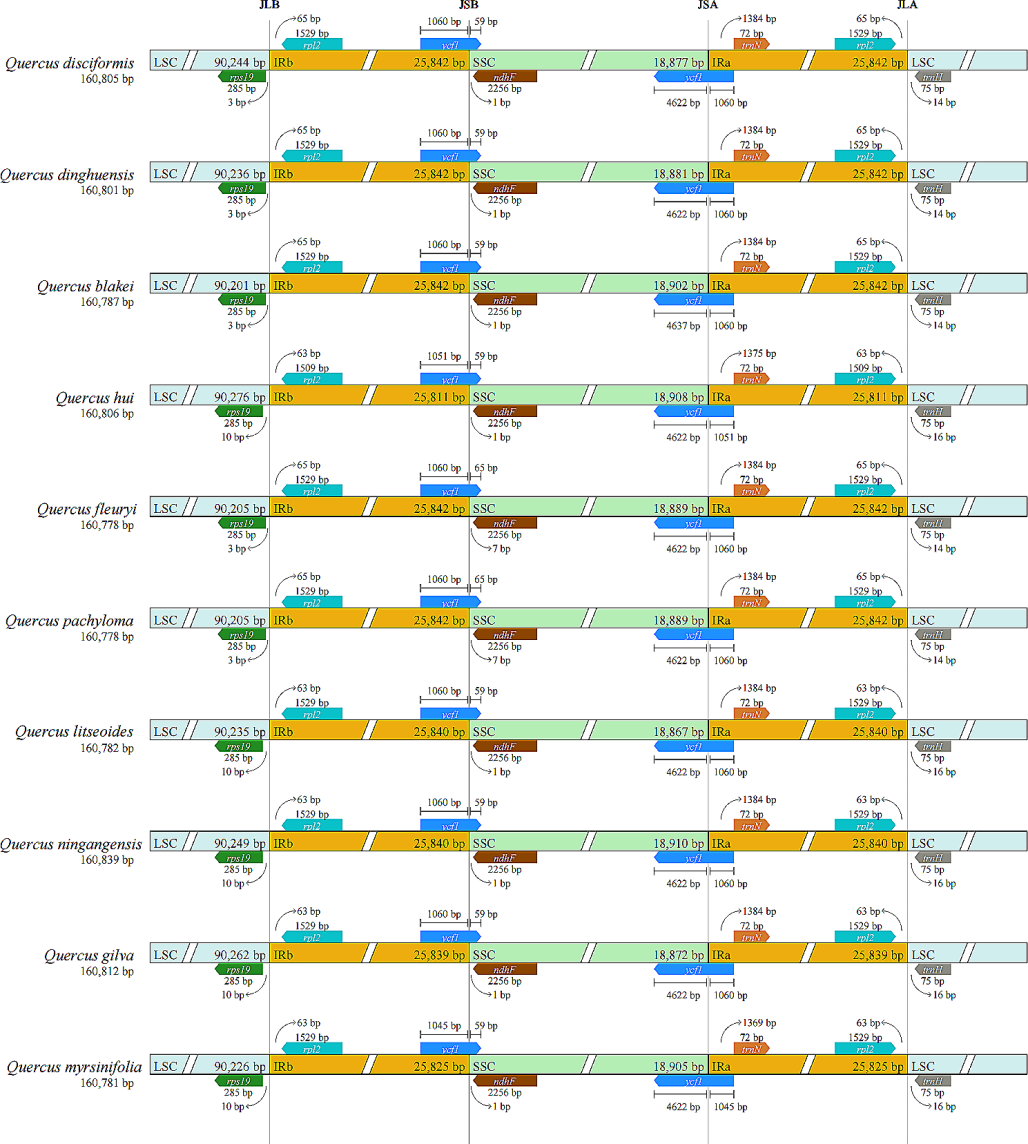
LSC, SSC and IR boundaries of the cp. genomes in 10 section *Cyclobalanopsis* cp. genome

A common feature of the chloroplast genomes in the ten species of section *Cyclobalanopsis* is the circular tetrad structure with four distinct boundaries: IRa-LSC, SSC-IRa, IRb-SSC, and LSC-IRb (Fig. 5). The genome sizes of these species were similar, with only minor differences; however, there were variations in the genes flanking these regions: *rps19*, *ndhF*, and *ycf1*. The study found that *rps19* was consistently located within the LSC region, but at varying distances from the LSC-IRb boundary—specifically at 4–11 bp. The *ndhF* and *ycf1* genes were positioned close to the SSC-IR boundaries. Found at the edge of the SSC region, the *ndhF* gene spanned 1,051 bp in *Q. hui* and 1,054 bp in *Q. myrsinifolia*, both within the IRa region. The *ycf1* gene, extending 1,060 bp, was located within the IRa region of a particular subgenus of section *Cyclobalanopsis*. Furthermore, an identical length fragment of *ycf1* was detected in the IRb region of the ten *Cyclobalanopsis* species, but it was considered a pseudogene (*ψycf1*).

#### Sequence divergence and hot spots

**Fig. 6:**
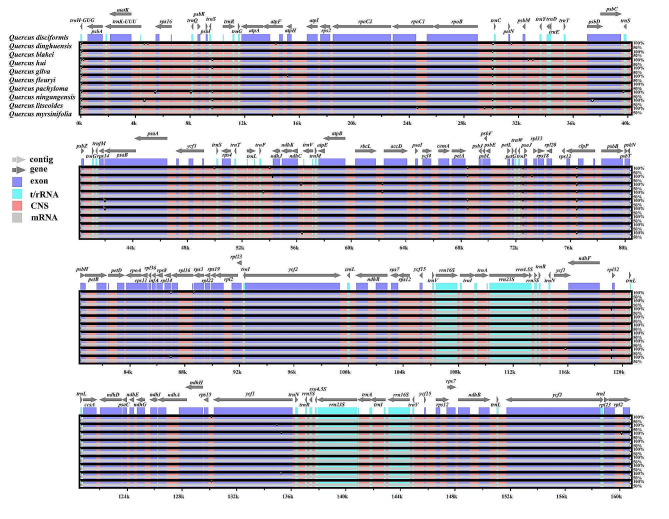
Comparison of cp. genomes of 10 section *Cyclobalanopsis* species

**Fig. 7:**
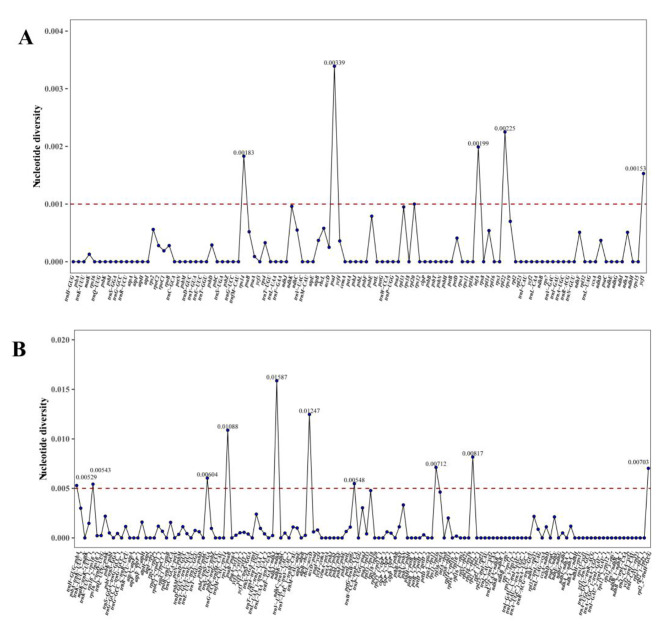
Comparison of nucleotide variability of common genes in 10 species of section *Cyclobalanopsis*

The current study utilized the Shuffle-LAGAN model in the mVISTA online software to analyze sequence variation in the cp. genomes among ten species of section *Cyclobalanopsis*, using the cp. genome sequence of *Q. kerrii* as a reference. The findings, as illustrated in Fig. 6, demonstrate that the coding regions of the cp. genomes from the ten *Cyclobalanopsis* species are highly conserved, while the rRNA genes exhibit minimal variation. However, certain disc*repan*cies persist across different cohorts, despite the typical conservation of the cp. genome in the section *Cyclobalanopsis*. Nucleotide polymorphisms in cp. genes were detected in the plants of the ten *Cyclobalanopsis* species. Figure 7 shows that sequence conservation in the intergenic spacer of the reverse repeat (IR) region is relatively higher than in the single-copy region. Based on these observations, the study screened the coding regions for the *rps14-psaB*, *ndhJ-ndhK*, *rbcL-accD*, and *rps19-rpl2_2 genes* (pi > 0.008), identifying them as potential DNA barcodes for investigating the genetic and phylogenetic relationships among species of the genus *Quercus*.

#### Phylogenetic analysis

**Fig. 8:**
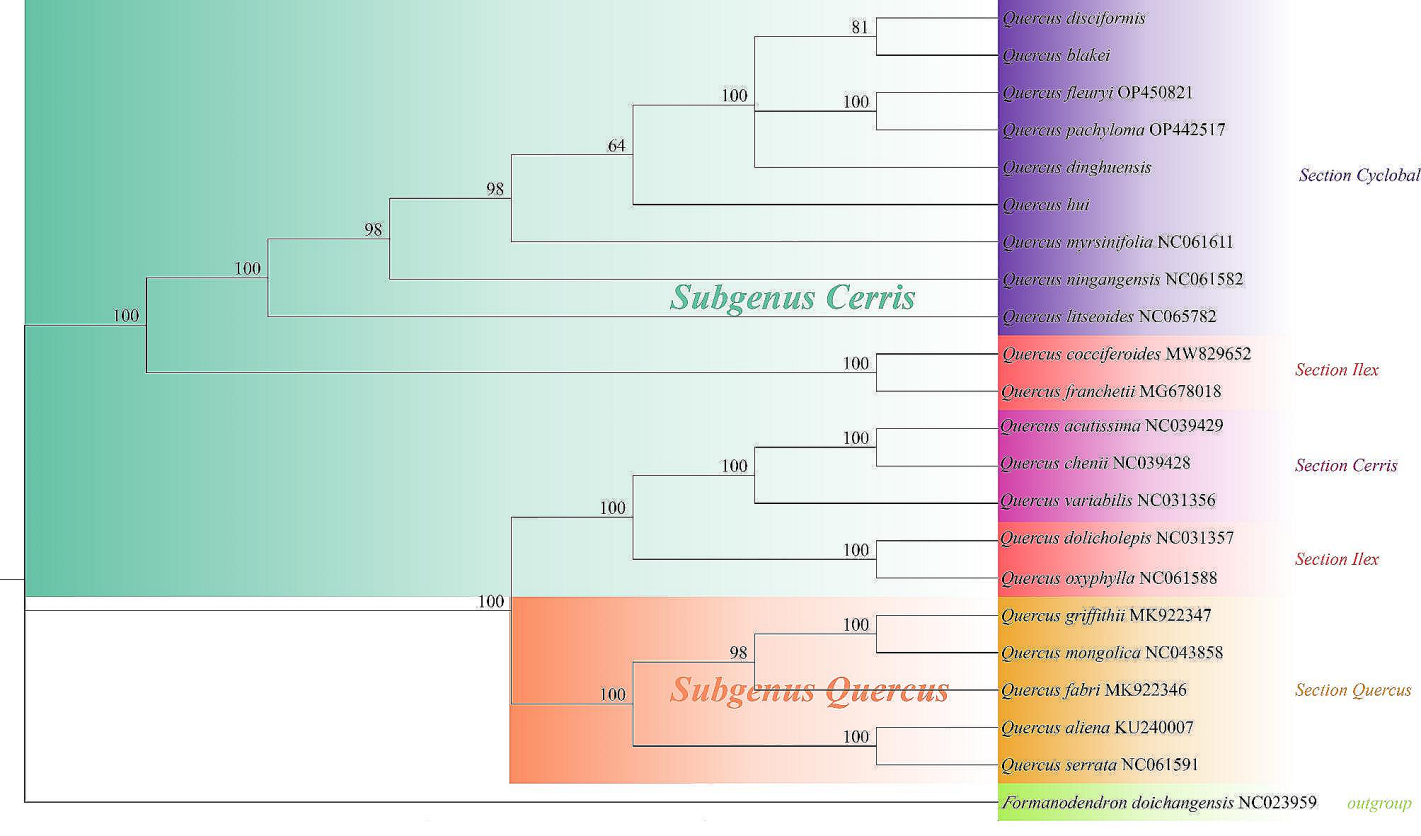
Phylogenetic relationships of 21 genus *Quercus* species inferred by ML method

This study utilize maximum likelihood (ML) to construct phylogenetic tree (Fig. 8). According to the phylogenetic tree, Formanodendron doichangensis is situated at the foundation of the phylogenetic tree and represents an initial divergent cluster within the *Fagaceae* family. The phylogenetic tree comprises two primary branches, namely *Subgenus Cerris* and *Subgenus Quercus*. The preceding category comprises of nine distinct species belonging to Section section *Cyclobalanopsis*, four species from Section *Ilex*, and three species from Section *Cerris*. The latter comprises five species from Section *Quercus*. Our analysis strongly supports *Q. dinghuensis*, *Q. disciformis*, *Q. blakei* and *Q. hui*, the four species, and *Q. fleuryi* and *Q. pachyloma* cluster together as a sister group that corresponds to previous classifications of the species. However, *Q. hui* appears slightly distant from the other five species. Within the *l*arger initial branch, the three Section *Cerris* species form a smaller branch with a 100/1.0 approval rating. However, the insertion of Section *Cerris* within Section *Ilex* results in the failure of Section *Ilex* to establish a monophyletic branch (All 21 species in Table 5).

**Table 5:** All 21 species constituting the phylogenetic tree and their Genbank numbers

Scientific Name	Genbank No.
*Quercus disciformis*	Newly sequenced species
*Quercus blakei*	Newly sequenced species
*Quercus fleuryi*	OP450821
*Quercus pachyloma*	OP442517
*Quercus dinghuensis*	Newly sequenced species
*Quercus hui*	Newly sequenced species
*Quercus myrsinifolia*	NC061611
*Quercus ningangensis*	NC061582
*Quercus litseoides*	NC065782
*Quercus cocciferoides*	MW829652
*Quercus franchetii*	MG678018
*Quercus acutissima*	NC039429
*Quercus chenii*	NC039428
*Quercus variabilis*	NC031356
*Quercus dolicholepis*	NC031357
*Quercus oxphylla*	NC061588
*Quercus griffithii*	MK922347
*Quercus mongolica*	NC043858
*Quercus fabri*	MK922346
*Quercus aliena*	KU240007
*Quercus serrata*	NC061591
*Formanodendron doichangensis*	NC023959

## Discussion

### The cp. genome of section ***Cyclobalanopsis***

The taxonomic system of the genus *Quercus* is globally recognized, yet analyses and reports of the complete cp. genomes within this framework have been limited [23, 32, 55, 57]. This study aims to address this gap by analyzing the cp. genomes of *Q. dinghuensis*, *Q. disciformis*, *Q. blakei*, and *Q. hui*. The entire sequences of the LSC, IR, and SSC regions of their cp. genomes were thoroughly analyzed and compared to examine their similarities. The results indicate that the plastid genome is highly conserved in terms of both structure and size. The cp. genomes of these four species comprise a total of 132 genes, which includes 87 protein-coding genes, 37 transfer RNAs, and 8 ribosomal RNAs. The ycf15 gene has been investigated in several studies for its potential functions. However, the status of ycf15 as a protein-coding gene in angiosperms is contentious. Although ycf15 is considered a pseudogene in most of the sequenced chloroplast genomes of the Fagales order, its presence has still been observed. It is important to note that the IR/SC boundary positions may change due to the contraction or expansion of the IR region, a common evolutionary phenomenon in phytoplasma genomes [18, 54]. Moreover, the GC content is an important indicator of phylogenetic relationships between species [40]. Therefore, an analysis of the overall GC content and the GC content in the IR and SC regions of four distinct *Quercus* species was conducted. The findings indicate that the overall GC content and the GC content within the IR and SC regions are largely consistent, with the GC content in the intergenic spacer (IGS) region exhibiting a statistically significant increase compared to the GC content in the large single-copy (LSC) region and the small single-copy (SSC) region. It is noteworthy that the ratio of AT to GC nucleotides is greater in all cp. genomes.

### Analysis of codon preference use

Codon usage preference is a critical aspect of biological evolution, influenced by various factors that affect the function of the genetic code. These factors include genome size, base mutations, genetic drift, natural selection, gene expression levels, and protein structure [3]. It is important to note that synonymous codons arise from mutations, and their relative usage can be quantified using the measure of relative synonymous codon usage (RSCU), which reveals variations in codon preference among different genes [45]. Through RSCU analysis, we have identified 61 codons, which show a clear preference for A/T-ending codons in these oaks. Interestingly, a similar trend was observed in the analysis of GC3 content, suggesting that the *Quercus* plants in our study exhibit a preference for codons ending in A/T. This preference for codon usage is a commonly observed phenomenon in angiosperms.

### Diversity of repetitive sequences and SSRs

Repetitive sequences play a crucial role in storing genetic information, influencing gene expression, and impacting the inheritance and evolution of plant species [41]. In our study, we examined ten different species within section *Cyclobalanopsis* and found that the number of simple sequence repeats (SSRs) ranged from 113 to 117. Among the types of SSRs, single nucleotide repeats were the most common, followed by dinucleotide and tetranucleotide repeats. The SSRs in section *Cyclobalanopsis* cp. genomes showed a high A/T base composition, indicating a preference for A/T bases. Notably, no hexanucleotide repeat sequences were detected, which is consistent with previous research on section *Cyclobalanopsis* [32, 55]. We also identified dispersed repeats in the ten distinct species of section *Cyclobalanopsi*s, with counts ranging from 40 to 41. These repeats were mainly composed of forward and palindromic sequences. However, variations in the number of tandem repeats among the species suggested different rates of mutational events.

### Genome variation and mutational hotspots

The accuracy of early phylogenetic analyses was improved by using variable regions or multiple DNA fragments, rather than relying solely on partial cpDNA sequences. However, the limited information in these sequences poses challenges in distinguishing between closely related taxa, especially within intra-species groups where taxonomic relationships are unclear. To address this issue, researchers have begun to use protein-coding regions and conserved sequences found in the cp. genome for more detailed phylogenetic analysis and variation studies among different species [41]. In the cp. genome, the phenomenon of single-nucleotide duplication (pi) is commonly observed and is considered indicative of mutational hotspots [21]. By examining the diversity in cp. genome sequences, researchers can identify genetic variations and pinpoint regions or genes with high levels of nucleotide diversity among different species. This method holds potential for creating new DNA barcodes, which are invaluable for classifying and differentiating various species.

### Phylogenetic relationships inference

China is recognized as the world’s second-largest center of diversity [6] and presents significant challenges in understanding the evolution of oak species. Taxonomic studies based on oak morphology are limited by convergent evolution and frequent hybridization among species. Despite these challenges, Deng Min established a taxonomic system for section *Cyclobalanopsis*. However, the only molecular phylogenetic analysis of the genus *Quercus* in China has depended on RAD-seq sequencing [12]. Most studies utilizing cp. genomes have successfully yielded high-resolution and well-supported phylogenetic trees, even in phylogenetically challenging plant taxa [15, 16, 31, 33]. In our study, we constructed a phylogenetic tree based on the alignment of chloroplast whole-genome sequences from *Q. disciformis*, *Q. dinghuensis*, *Q. blakei*, and *Q. hui*, along with 17 other closely related species of section *Cyclobalanopsis*. The analysis reveals that these four species form a sister clade, with *Q. hui* being the first to diverge from the group, and *Q. disciformis* and *Q. blakei* emerging as the closest relatives. This suggests a higher degree of collinearity in their evolution. The pollen morphology of these oaks, characterized by echinate and aggregate baculate ornamentation [10], supports this conclusion. An analysis of branch characteristics, such as leaf epidermis and structure, classified *Q. hui* and *Q. dinghuensis* as simple hairy types, with *Q. hui* being the earliest divergent species. Conversely, *Q. disciformis* and *Q. blakei* were classified as complex hairy types and identified as the closest relatives. These findings are corroborated by numerical taxonomy and principal component analysis (PCA) [9, 22, 34]. Moreover, our research indicates that section *Cerris* is nested within section *Ilex*, challenging the concept of a monophyletic branch for section *Ilex*. Prior studies on the evolution of the *Quercus* genus have documented hybridization events between sections *ilex* and *Cerris*, including shared plastid haplotypes, the non-monophyly of section *Ilex* in barcoding studies, and the admixture of certain species within sections *Ilex* and *Cerris*. Such observations may be explained by incomplete lineage sorting and introgression between the two sections. The complexity of phylogenetic studies on the genus *Quercus* can be attributed not only to extensive cross-introgression but also to discrepancies among relationships based on different plastid and nuclear markers. In line with previous taxonomic systems that used nuclear markers for the global genus *Quercus* [20], our research classifies the Chinese genus *Quercus* into four well-supported sections: *Ilex*, *Cerris*, *Cyclobalanopsis*, and *Quercus*. The study provides strong support for the inclusion of the monophyletic sections *Cyclobalanopsis* and *Cerris* within section *Ilex*. However, nuclear data did not confirm a monophyletic section *Ilex* [39, 52], possibly due to cpDNA capture, which is the phenomenon where hybridization or gradual infiltration results in the transfer of chloroplasts from one species to another, leading to variations in plastid genotypes that are stably inherited across generations. Although genomic information may offer a potential solution to species classification issues within the genus *Quercus*, current research shows that the cp. genome constitutes just a part of the plant genome, emphasizing the complexity of this issue. Therefore, the development of advanced genetic methods for phylogenetic or population inference is expected to advance our understanding of the evolution of the genus *Quercus*.

## Materials and methods

### Plant materials and cp. DNA extraction

Specimens of *Q. disciformis* and *Q. dinghuensis* were collected from the South China Botanical Garden, Chinese Academy of Sciences (at an altitude of 532 m, 113°72’37’’ E, 23°33’28’’ N), and Q. blakei and Q. hui were collected from the Nanning Arboretum, Guangxi Province, China (at an altitude of 1204 m, 108°31’35’’ E, 22°70’94’’ N). These specimens were identified by Associate Professor Zhang Xuemei of China West Normal University as Q. *disciformis*, *Q. dinghuensis*, *Q. blakei*, and *Q. hui*. The leaves were cleaned with damp gauze cloth and then stored in sealed containers with discolored silica gel. Subsequently, the leaves were dried and stored at -80 degrees Celsius until analysis. The botanical specimens are preserved in the Herbarium at the College of Life Sciences, China West Normal University (SITC). Total genomic DNA was isolated using the TruSeq DNA Sample Preparation Kit (Illumina, San Diego, CA, USA). The specimens were assigned the following numbers: LY221001 (Quercus disciformis), LY221002 (Quercus dinghuensis), LY221003 (Quercus blakei), and LY221004 (Quercus hui). Gel electrophoresis was performed to determine the DNA concentration and purity. Double-ended sequencing of the extracted DNA was conducted using Illumina NovaSeq platforms. After removing low-quality reads with FASTQ [8] software, clean data for subsequent analysis, including approximately 5 GB of high-quality sequence, were obtained. The extraction and sequencing of cp genomic DNA were carried out by Beijing Berry Genomics Company. Additionally, 17 other cpDNA sequences were retrieved from the NCBI for comparative analysis.

### Cp genome assembly and genes annotation

The filtered sequence from GetOrganelle [24] was used to assemble the cp. genome, with *Q. kerrii* (sequence number: OP679796.1) serving as the reference sequence. The assembled cp. genome was then annotated using GAVAS2 [51]. To ensure accuracy, the annotation process included manual corrections using Geneious [26] software, which incorporated start and stop codon positions, as well as intron and exon boundaries. Comparative analysis with relevant species was also conducted to enhance the precision of the annotation outcomes. The resulting annotated cp. genome sequences were submitted to the U.S. National Center for Biotechnology Information (NCBI) Database under the accession numbers OQ596300-OQ596303. Additionally, the annotation file was uploaded to the online site OGDRAW [36] to generate four complete genome maps of section *Cyclobalanopsi*s of the cp.

### Comparison of related cp. genomes

This investigation aimed to compute the lengths and GC content of the Small Single Copy (SSC), Large Single Copy (LSC), and Inverted Repeat (IR) regions in the cp. genomes of four section *Cyclobalanopsis* species, using Geneious software [26]. Additionally, comparative analysis was performed on six cp. genomes obtained from NCBI, identified as closely related. These genomes include *Quercus ningangensis* (NC_061582), *Quercus litseoides* (NC_065782), *Quercus gilva* (MG678009), *Quercus myrsinifolia* (NC_061611), *Quercus fleuryi* (OP450821), and *Quercus pachyloma* (OP442517).

#### SSR and sporadic repeat sequences

The study conducted a comprehensive examination of scattered repetitive sequences in the cp. genomes of ten different section *Cyclobalanopsis* species using the online software REPuter [30]. The analysis investigated forward (F), reverse (R), palindrome (P), and complementary (C) repeats. Specific parameters were employed, including a minimum repeat length of 30 and a Hamming distance of 3, requiring a minimum similarity of 90% between repeat sequences. The default settings were used for the remaining parameters, with a total of 1000 parameters established. For the analysis of Simple Sequence Repeats (SSRs), MISA [4] software was utilized. Different thresholds were applied for various nucleotide repeats, including parameters of 1–10 (single nucleotide repeats occurring at least ten times), 2–5, 3–4, 4 − 3, 5 − 3, and 6 − 3 for SSR analysis. All other parameters remained at their default settings. Manual validation was performed on all analyzed repetitions, and any redundant results were eliminated.

#### Codon bias analysis

This study conducted an initial screening of 52 unique non-repetitive sequences, each exceeding 300 base pairs and including the ATG start codon, to prepare for further analysis. We used the CodonW 1.4.2 program [46] to calculate various codon usage indices and base composition statistics for each coding sequence. The analysis involved calculating metrics related to codon usage, such as relative synonymous codon usage (RSCU), codon adaptation index (CAI), effective number of codons (ENC), codon bias index (CBI), frequency of optimal codons (FOP), ENC values, RSCU values, and the probabilities of each base occurring at the third position of a codon. The GC1, GC2, and GC3 contents of the coding sequences were determined using EMBOSS software [48].

#### Sequence variation of cp. Genome

To assess gene rearrangements and boundaries within the large single copy (LSC), small single copy (SSC), and inverted repeat (IR) regions of ten section *Cyclobalanopsis* species, the researchers utilized the IRscope [2] online tool to generate horizontal visualizations. They conducted a comparative analysis of sequence variations across ten genomes using the MVISTA software in shuffle-LAGAN mode. The MVISTA program is accessible at the following URL: (http://genome.lbl.gov/vista/mvista/submit.shtml). They assessed nucleotide variation within cp. genomes by screening for sites with high variability using DNAsp6 software [50], based on the nucleotide diversity index (π).

### Phylogenetic analysis of genus ***Quercus***

The study employed maximum likelihood (ML) and Bayesian inference (BI) methods to construct a phylogenetic tree that included four sections of genus *Quercus* endemic to China, encompassing a total of 21 species. *Formanodendron doichangensis* was employed as the outgroup in the analysis. The comparison of sequences was conducted through the utilization of MAFFT [25] v7.467, followed by sequence clipping in mega for further refinement. The construction of ML trees was carried out utilizing MEGAX [29], employing the General Time Reversible model model as recommended, and setting the bootstrap to 1000 iterations. The BI tree was generated through the utilization of MrBayes v3.2.7 [49]. The MCMC algorithm was employed for an overall amount of 2 million generations, with a sampling frequency of once every 500 generations. The burn-in phase, which constituted the initial 25% of the aging generation samples, was excluded. Subsequently, a coherent tree was constructed and posterior probabilities were computed using the remaining samples. The optimal alternative model was determined by illustrating and evaluating the phylogenetic tree through the utilization of Figtree [47]v 1.4.3. 17. genus *Quercus* species’ cp. genomes were publicly released on NCBI, and four section *Cyclobalanopsis* species’ cp. genomes were assembled in this study. These were chosen as outer taxa for *Formanodendron doichangensis*. Phylogenetic trees were constructed using both maximum likelihood (ML) and Bayesian (BI) methods to investigate the relationship between genus *Quercus* species in China.

## Conclusion

The current investigation applied high-throughput sequencing technology to explore the chloroplast genomes of *Q. dinghuensis*, *Q. disciformis*, *Q. blakei*, and *Q. hui.* It also analyzed the structural characteristics of their chloroplast genomes. Given the limited research on section *Cyclobalanopsis* and the challenges in studying genus *Quercus*, this study makes a significant contribution by analyzing the chloroplast genome of section *Cyclobalanopsis* and revealing its structural and general variations. Furthermore, the findings provide an initial understanding of the genus *Quercus* in China and offer a new reference for species identification and genetic variation analysis at the population and individual levels.

## Data Availability

The complete chloroplast genomes of, Q. disdisformis and Q. dinghuensis, Q. blackkei and Q. hui were submitted to the NCBI database (https://www.ncbi.nlm.nih.gov/) with GenBank accession numbers OQ596300 (Quercus disciformis), OQ596301 (Quercus dinghuensis), OQ596302 (Quercus blackei) and OQ596303 (Quercus hui). All other data and material generated in this manuscript are available from the corresponding author upon reasonable request.
